# Genetic ancestry and population structure of vaccinia virus

**DOI:** 10.1038/s41541-022-00519-4

**Published:** 2022-08-11

**Authors:** Cristian Molteni, Diego Forni, Rachele Cagliani, Mario Clerici, Manuela Sironi

**Affiliations:** 1 IRCCS E. MEDEA, Bioinformatics, Bosisio Parini, Italy; 2 University of Milan, Milan, Italy; 3 Don C. Gnocchi Foundation ONLUS, IRCCS, Milan, Italy

**Keywords:** Biologics, Immunology

## Abstract

Vaccinia virus (VACV) was used for smallpox eradication, but its ultimate origin remains unknown. The genetic relationships among vaccine stocks are also poorly understood. We analyzed 63 vaccine strains with different origin, as well horsepox virus (HPXV). Results indicated the genetic diversity of VACV is intermediate between variola and cowpox viruses, and that mutation contributed more than recombination to VACV evolution. STRUCTURE identified 9 contributing subpopulations and showed that the lowest drift was experienced by the ancestry components of Tian Tan and HPXV/Mütter/Mulford genomes. Subpopulations that experienced very strong drift include those that contributed the ancestry of MVA and IHD-W, in good agreement with the very long passage history of these vaccines. Another highly drifted population contributed the full ancestry of viruses sampled from human/cattle infections in Brazil and, partially, to IOC clones, strongly suggesting that the recurrent infections in Brazil derive from the spillback of IOC to the feral state.

## Introduction

Smallpox, caused by variola virus (VARV, genus *Orthopoxvirus*, family *Poxviridae*), was the first human infectious disease to be eradicated and, to date, the only one. VARV was also the first infectious agent against which a vaccine was developed. Indeed, vaccination was introduced in 1796 by Edward Jenner, who showed that humans could be protected against smallpox by inoculation of material deriving from animal lesions^1^. It was initially assumed that the vaccine developed by Jenner was based on cowpox virus (CPXV), another member of the *Orthopoxvirus* genus. However, it is now clear that the virus used to immunize against smallpox, now referred to as vaccinia virus (VACV), is more closely related to horsepox virus (HPXV)^1,2^. Thus, the biological origin of the smallpox vaccine is unsure and the situation is further complicated by the fact that neither cows nor horses are likely to represent the original hosts of CPXV and HPXV^3^. As a consequence, the natural host of VACV is unknown. Notably, VACV infections of animals (primarily cattle in South America and water buffalo in Asia) and humans have been repeatedly reported^3^. It is still unsure whether such infections derive from viruses that originated from the spillback of vaccine strains or if they represent natural VACV populations circulating in an unknown wild host^3^.

In the years that followed Jenner’s discovery, different inocula used as vaccinating agents were repeatedly passaged in humans and, later, in animals. The most common practices involved virus inoculation in calves, heifers, rabbits, sheep, mice, or chick embryos^2,4^. The inocula were also transported and distributed throughout the world to be used in local vaccination campaigns. For instance, at the end of the 19th century, the New York City Board of Health (NYCBH) was producing the smallpox vaccine from inocula originally transported from England and Cuba^2^. Derivatives of the NYCBH vaccine were distributed to other laboratories and received different names. Supposedly, the Dryvax vaccine, which is a mixture of different clones and was widely used in the USA, was derived from the NYCBH stock. However, historical records suggest that the Beaugency lymph, used to seed vaccine production in France, was also (or mainly) used to manufacture Dryvax^2^. The Beaugency lymph reached many countries, including Brazil, where it was used to derive the IOC (Institute Oswaldo Cruz) vaccine^2^. The WR (Western Reserve) and IHD (International Health department) strains are instead thought to derive from the NYCBH stock^2^. In particular, IHD underwent a long passaging procedure and later reached Japan, where the IHD-J and IHD-W strains were derived^5,6^.

Other vaccines, including Lister, Tian Tan, and Tashkent found diffusion in Europe and Asia. Their origin and passage histories are often unknown or murky. As an example, the Tian Tan vaccine, which was widely used in China, was reportedly isolated in Beijing’s Temple of Heaven from a patient with smallpox and subsequently passaged in cows, monkeys, and rabbits. However, the host range of VARV is known to be restricted to humans and the sequencing of Tian Tan clones clearly indicated that the virus used for vaccination was VACV and not VARV^7,8^. Thus, the origin of the Chinese vaccine is unknown.

Over the years, a number of VACV clones and strains have been sequenced. These include historical samples, such as a stock manufactured in 1902 by the Philadelphia company H.K. Mulford^9^ and vaccination kits preserved at the Mütter Museum of the College of Physicians of Philadelphia, dating to the mid-to-late nineteenth century^10^. Phylogenetic analyses showed that these historical vaccine genomes cluster with a HPXV strain, isolated from a horse in an 1976 outbreak in Mongolia^10,11^. The availability of several sequenced VACV genomes thus allows analysis of their evolutionary histories. This is relevant from an historical perspective, but it is also topical, as zoonotic orthopoxviruses such as CPXV and monkeypox virus (MPXV) are increasingly reported as causes of human disease^12,13^. In particular, an unprecedented MPXV multi-country outbreak outside the endemic area in Africa has been expanding since the first cases were reported in the UK, in May 2022^14^. Because smallpox vaccination with VACV provides cross-protection against multiple orthopoxviruses, the raise in MPXV and CPXV human cases is likely to be partially the result of discontinuation of routine smallpox vaccination^15^. Thus, a vaccine based on the modified vaccinia virus Ankara (MVA) is currently being offered to subjects at risk of exposure and to healthcare workers in the UK^16,17^.

## Results

### Genetic diversity of VACV

**Fig. 1:**
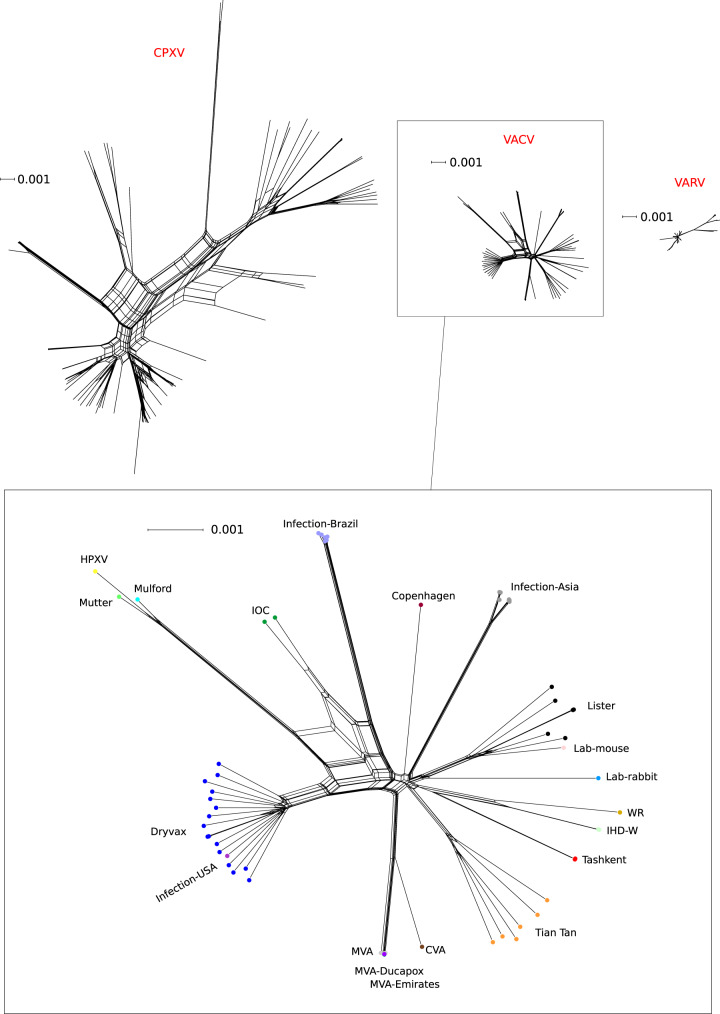
Genetic variability within different orthopoxvirus species. Neighbor-net split network of 90 CPXV, 50 VARV, and 64 VACV genome sequences; the three networks are plotted at the same scale (expressed as substitutions/site). An enlargement of VACV genomes is also provided, with samples shown as dots and colors representing group membership.

We obtained a list of 64 complete or almost complete VACV genomes from public databases (Supplementary Table 1). These include the Mütter Museum and Mulford historical samples, several Dryvax, Lister, and Tian Tan clones, the single HPXV genome, the chorioallantois vaccinia virus Ankara (CVA, the parental virus of MVA), and other vaccine strains (e.g., WR, IOC, IHD-W). The dataset also comprises isolates from human, cattle, or buffalo infections in Brazil and Asia, as well as some strains derived from rabbit or mouse outbreaks in laboratory settings. Generation of a neighbor-net split network indicated clustering by genome origin (Fig. 1). In line with previous observations, a virus sequenced from mice during a lab outbreak and a human infection in the USA clustered with Lister and Dryvax, respectively^18,19^.

To compare the genetic diversity of VACV to other orthopoxviruses deriving from natural transmissions, neighbor-net split networks were also generated for 50 modern VARV sequences and 90 CPXV sequences (Supplementary Table 2). VACV was definitely more diverse than VARV, but much less than CPXV (Fig. 1).

### Genetic relationships among VACV genomes and population structure

**Fig. 2:**
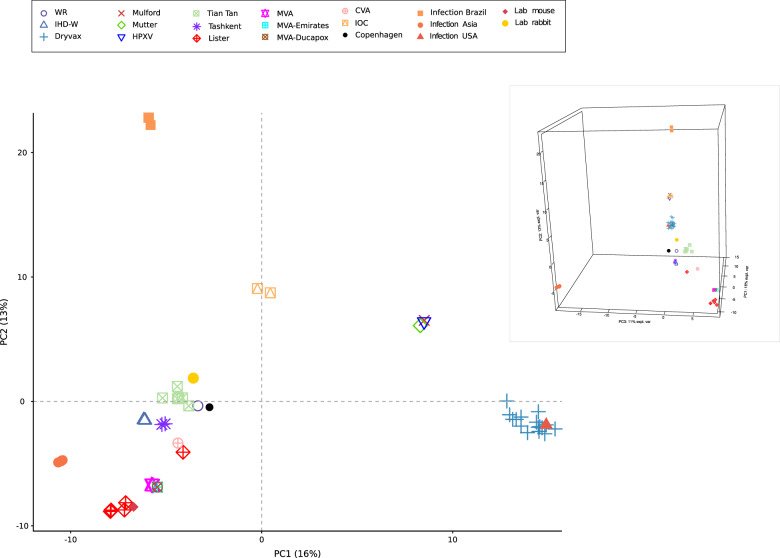
PCA of vaccinia virus genomes. A principal component analysis of VACV samples is shown. Each VACV group/type is colored and displayed with a different symbol, as described in the legend. The two first PCs are plotted in the large figure. In the insert, the two axes (PC1 and PC2) are rotated to visualize the contribution of PC3.

To gain insight into the genetic relatedness of VACV genomes, we applied principal component analysis (PCA). The first two components clustered sequences based on origin or type (e.g., most vaccine strains were grouped by vaccine type). In line with previous phylogenetic analyses, HPXV, Mulford and Mütter Museum sequences clustered together (Fig. 2). The first PC clearly separated most American strains (Dryvax clones, Mulford and Mütter Museum sequences) from all other strains, with IOC having an intermediate placement. The second component separated the Brazilian infections (both cattle and human) from the other sequences, with IOC and HPXV/Mütter/Mulford genomes in intermediate position (Fig. 2). In line with previous phylogenetic analyses, two strains thought to be derived from the NYCBH stock, namely IHD-W and WR, clustered with the European/Asian sequences^2,6,7^. IOC sequences were the closest to the viral genomes deriving from the Brazilian infections (Fig. 2). The third PC contributed to the further separation of Eurasian sequences, while adding little to the separation of the American strains. The viruses responsible for human/buffalo infections were placed distant from all other genomes. The divergent Lister clone is a Lister-derived recombinant oncolytic virus^20^ (Fig. 2).

To gain further insight into the structure of VACV populations, we used the program STRUCTURE, which was widely applied to infer ancestry and admixture patterns^21–23^. STRUCTURE relies on a Bayesian statistical model for clustering genotypes into populations without prior information on their genetic relatedness. The program can identify distinct subpopulations (or clusters, K) that compose the overall population. Subpopulations can then be related to specific features such as origin, genotype classification, or phenotype. We thus considered that this approach might provide insight into the ancestry and evolutionary history of VACV genomes.

Because STRUCTURE is ideally suited for weakly linked markers, we first analyzed the level of linkage disequilibrium with LIAN v3.7, which tests the null hypothesis of linkage equilibrium across loci^24^. Statistically significant LD was detected (Monte Carlo simulations, 1000 repetitions, *p* < 10^−3^), but the standardized index of association (I_A_^S^) resulted equal to 0.038. This value indicates weak LD, most likely as a cause of recombination and other processes, and warrants the application of STRUCTURE models. Specifically, we used the linkage model with correlated allele frequencies, which assumes that discrete genome “chunks” were inherited from K ancestral populations^22^.

**Fig. 3:**
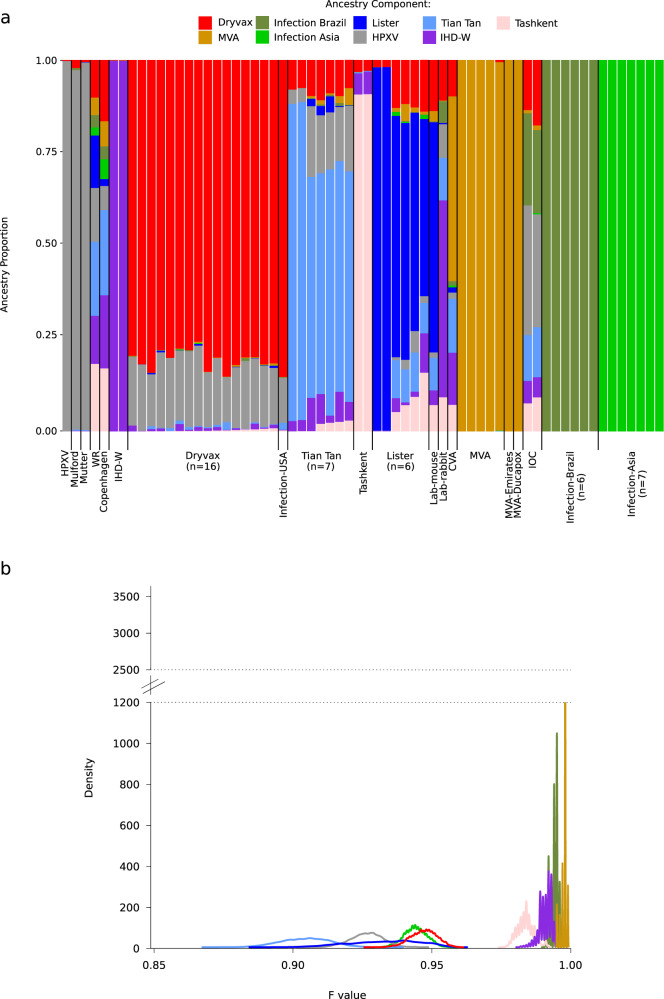
Population structure of vaccinia virus. **a** Bar plot representing the proportion of ancestral population components for *K* = 9. Each vertical line represents a VACV genome and it is colored by the proportion of sites that have been assigned to the nine populations by STRUCTURE. Ancestry components are named based on the genomes where they are more prevalent. **b** Distributions of F values for the nine populations. Colors are as in panel a. Y axis is cut to better display all density distributions.

To estimate the optimal number of subpopulations in the VACV dataset, STRUCTURE was run for values of K from 1 to 14. The ΔK method yielded a major peak at *K* = 9 (Supplementary Fig. 1). Analysis of ancestry components was thus performed for 9 subpopulations and by plotting genomes according to their origin or type (Fig. 3a). Results showed a very good clustering by origin, as well as concordance with the neighbor-net split network and PCA analyses. Thus, the nine subpopulations roughly identified the HPXV/Mütter/Mulford strains, Dyvax sequences (together with the USA infection), Lister strains (together with the lab mouse infection), Tian Tan, IDH-W, MVA, and Tashkent clones. Two additional populations identified the Brazilian and Asian infections (Fig. 3a). All these sequences formed distinct clusters in the network and PCA analyses (Figs. 1 and 2). Evidence of extensive admixture was observed for other VACV genomes (i.e., IOC, WR, Copenhagen, CVA, and the lab rabbit strain) (Fig. 3a) that formed distinct branches in the neighbor-net split network and were separated in the PCA (Figs. 1 and 2).

We next used the linkage model in STRUCTURE to estimate the level of drift of each subpopulation from a hypothetical common ancestral population. Specifically, we estimated the F parameter, which represents a measure of genetic differentiation between populations based on allele frequencies. Results indicated that the lowest drift was experienced by the two subpopulations that account for the largest ancestry component of Tian Tan genomes and for the full ancestry of HPXV/Mütter/Mulford sequences (hereafter referred to as subpopulations HPXV and Tian Tan) (Fig. 3b). In line with the PCA analysis, subpopulation HPXV accounted for variable proportions of the ancestry of the IOC and Dryvax clones, of Tian Tan strains, and of the WR sequence (Fig. 3a). The subpopulation highly represented in Tian Tan strains also contributed to the WR genome, as well as to most Lister and IOC clones, to the Copenhagen strain, and to the ancestry of CVA (Fig. 3a). Some level of uncertainty in the estimation of F was observed for one of the populations showing low drift (Fig. 3b). This component accounted for the major ancestry of Lister clones, and it was mainly restricted to Lister samples, with the exception of a contribution to WR (Fig. 3a). Two populations with slightly higher drift represented the major ancestry of Dryvax clones and the full ancestry of buffalo and human infections sampled in Asia (Fig. 3b). Whereas the Dryvax component was evident in several other strains, the human/buffalo infection component was virtually restricted to the Asian strains (Fig. 3a).

Finally, four subpopulations were found to have experienced very strong drift (Fig. 3b). One of these almost fully contributed to the Tashkent ancestry and minimally to other genomes. Two other highly drifted subpopulations accounted for the full ancestry of MVA and IHD-W sequences (Fig. 3a). This is in very good agreement with the notion that MVA and IHD-W are among the most passaged VACV strains. Interestingly, the fourth high-drift population contributed the full ancestry of viruses responsible for the Brazilian human and cattle infections. Other than these sequences, this component was present at appreciable frequency only in the IOC clones, in line with the PCA analysis (Figs. 2 and 3a). These data strongly support the view that the recurrent infections in Brazil derive from the spillback of IOC to domestic and possible wild hosts.

**Fig. 4:**
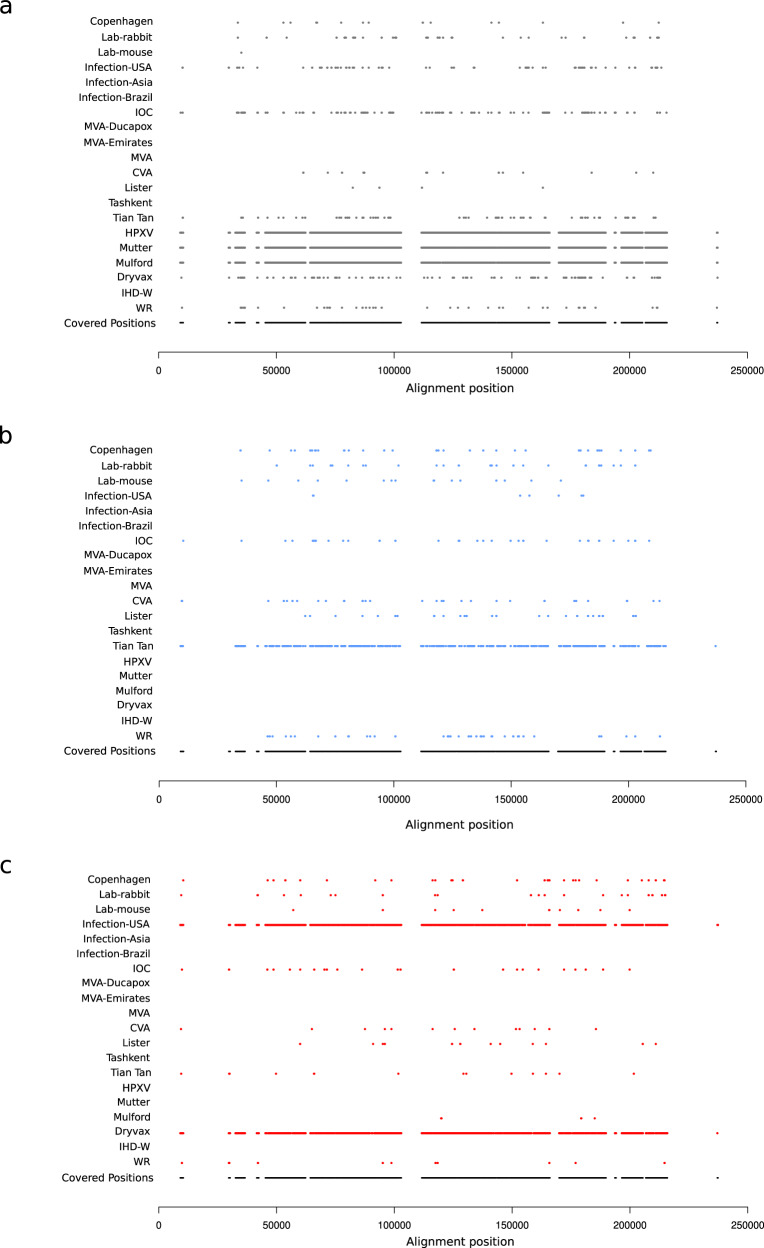
Ancestral component probabilities at nucleotide resolution. Site by site probability of ancestry components for a subset of VACV genomes. Each line represents a randomly selected virus for each of the clones/populations of VACV; each dot represents a PI site with a probability >0.75 to derive from the **a** HPXV, **b** Tian Tan, and **c** Dryvax ancestry component. Colors are the same as in Fig. 3. The bottom line represents the position of all PI sites (as black dots) analyzed in this study. Positions refer to the genome alignment. Regions that are not covered by PIs are highly dynamic and sequence information was present for less than 90% of sequences.

To gain further insight into the ancestry contribution to VACV genomes, we exploited the site-by-site inference in STRUCTURE, which allows population-of-origin assignment for individual variants. The analysis was performed for the four components (HPXV, Tian Tan, Dryvax, and Lister) that are shared across several VACV genomes (Fig. 4 and Supplementary Fig. 2). The HPXV component is the most widely shared among the VACV genomes we analyzed. Clearly, this component contributes to the almost full ancestry of HPXV, Mütter and Mulford sequences (Fig. 4). In all other instances, irrespective of their origin, contributing HPXV variants are not clustered but rather scattered throughout the genome. This observation is also true for the Tian Tan, Lister, and Dryvax components (Fig. 4 and Supplementary Fig. 2). Thus, the admixture observed in VACV genomes is not the result of the exchange of large genomic fragments. Conversely, these patterns of intermixed small blocks suggest a scenario of recombination of short genomic regions or evolution by mutation from a common population(s), or both.

### Recombination and mutation in VACV evolution

To analyze the relative contribution of recombination and mutation in the evolution of VACV strains, recombination was inferred genome-wide for the 64 genomes using ClonalFrameML^25^. In total, 988 recombination events were detected along the branches of the inferred phylogeny. The average length of the recombinant segments was 36.9 bp; the average divergence between donor and recipient was 0.175. The genome-wide rate of recombination to mutations (R/theta) was estimated to be equal to 0.07, meaning that mutations happened about fourteen times more often than recombination events. Overall, the relative effect of recombination to mutation (r/m) was 0.46. So, nucleotide changes in the VACV phylogeny were twice more likely to arise from mutation than by recombination.

## Discussion

The events that unfolded since Jenner’s seminal intuition until the eradication of smallpox have no equal in the annals of infectious diseases and of medicine at large. The evolutionary history of VACV and its convolutions are intimately linked to those events. VACV reached every corner of the world in the form of vaccine inocula, but its ultimate origin and natural host(s) remain unknown. Thus, the genetic makeup of VACV is the result of artificial virus growth, passaging, selection, and migration. Most likely, extant VACV strains and clones derive from a limited number of seed viruses^2,6^, a situation that clearly introduced founder effects and limited genetic variability. The genetic diversity of VACV is intermediate between VARV and CPXV, two viruses with very different host range and epidemiology. Like CPXV, VACV can infect many different hosts, suggesting that, had it evolved naturally, it might display a diversity comparable to that of CPXV. However, the possible extinction of natural VACV lineages implies that important differences with CPXV might exist. Indeed, it is unsure whether any natural VACV sequence has ever been sampled, as even the HPXV strain might represent a vaccine escapee to the feral state. Nonetheless, based on the pattern of gene inactivation, it has been suggested that HPXV or a HPXV-like virus was the progenitor of several early inocula that were turned into vaccines. Our data strongly support this hypothesis, as the ancestry of HPXV, as well as those of the Mütter Museum and Mulford vaccines, are fully accounted for by a population showing low drift. The HPXV ancestry component is also one of the most widespread among the VACV genomes we analyzed. In particular, it contributed a significant ancestry portion in IOC vaccines, which are thought to be derivatives of the Beaugency lymph^2^. This strengthens the tie between this latter and the HPXV/Mulford/Mütter cluster^2,10^. These data are also in agreement with the view that Dryvax, also showing a HPXV ancestry component, was mainly derived from the Beaugency lymph^2^. Nonetheless, the HPXV population is not the one showing the lowest F values. The Tian Tan component has lower drift, although the F distribution partially overlaps with that of HPXV, as well as with the Lister component. Reportedly, the Tian Tan strain was first isolated in 1923, but its origin is unknown. In line with previous phylogenetic analyses^7,8^, we found the Tian Tan component to be also present in the Copenhagen, Lister and WR sequences, as well as in the two IOC strains. Overall, it is tempting to speculate that HPXV-like and Tian Tan-like viruses, whether they were part of the same or of distinct populations, have served as the ancestors of most vaccine lineages that subsequently evolved independently.

Our results showing strong drift of the MVA and IHD-W populations is in good agreement with the passage history of these vaccine strains. Indeed, MVA was derived from CVA by more than 570 passages in chicken embryo fibroblasts. As a result, MVA acquired a restricted tropism for avian cells^26^. The ancestor of IHD-W underwent several rounds of intracerebral infection in mice and chorioallantoid membranes before reaching Japan^5,6^. High drift was also observed for the two Tashkent clones. Tashkent, whose use in humans was discontinued due to severe adverse effects, is generally considered a relatively old and pristine vaccine^6^. However, its origin is unknown, and our analyses suggest that it experienced a level of drift comparable to MVA and IHD-W, possibly indicating a long passage history or a strong bottleneck resulting from other effects. Notably, the fourth population that experienced strong drift accounts for the ancestry of viruses responsible for cattle and human infections in Brazil (Br-VACV). This component is also present in the IOC clones, but not in other VACV sequences. Thus, these data are in line with the PCA results and with previous indications that Br-VACV sequences are more closely related to IOC than to HPXV^1,27^. These observations strongly suggest that Br-VACV derived from the escape of an IOC-like vaccine strain and that the spillback caused a bottleneck in the viral population. It should however be mentioned that VACV infections in Brazil are caused by viruses belonging to two distinct lineages (referred to as Br-VACV group 1 and Br-VACV group 2)^28^. We only analyzed Br-VACV group 1 (which includes Cantagalo virus and Serro 2), as no complete viral genome is available for group 2. Thus, the history of Br-VACV is necessarily more complicated than the one we can reconstruct, and the sequencing of Br-VACV group 2 genomes will be necessary to reach to the full scenario. Even more enigmatic is the situation of the Asian infections. The ancestry component accounting for the VACV genomes sampled in Asia is in the low range of F values and is virtually absent from any other genome analyzed herein. These data therefore do not support the view that these infectious strains derive from the Lister vaccine and, in PCA, they were similarly distant from many Eurasian vaccines. Thus, if these infections represent another spillback event, the source vaccine strain may have remained unsampled. Alternatively, these infections, which were first described in 1934, might be caused by natural viruses circulating in an unknown reservoir(s)^3^.

Previous studies that analyzed recombination patterns in Dryvax genomes^29,30^ suggested that recombination contributed to the diversification of VACV clones and described a patchy pattern consistent with the exchange of short genomic fragments. Our analysis with ClonalFrameML across the entire VACV phylogeny is consistent with this possibility and we found that the average length of recombining fragments is less than 40 bp. Indeed, the same analysis revealed that mutation played a more relevant role than recombination in the evolution of VACV. This might seem counter-intuitive, as the production of VACV in artificially infected animals and cells probably often resulted in high multiplicities of infection, which might promote recombination. However, microscopy studies have shown that the mode of orthopoxvirus replication within infected cells creates physical constraints that reduce opportunities for forming recombinants^30,31^. Also, it should be mentioned that recombination events that occurred between distantly related genomes are easier to detect than events involving close relatives. In particular, ClonalFrameML is based on a model of extra-population recombination, although it can also detected a large proportion of intra-population recombination^32^. Thus, the program might have missed some events that occurred among closely related VACV genomes (e.g., those that may have occurred among the clones of a vaccine stock).

Finally, we should add that most of our analyses were based on PI sites for which at least 90% of sequences had non-missing information. Whereas this had the purpose of excluding poorly aligned regions, we certainly missed information from the more dynamic portions of VACV genomes, which evolve by gene gains and losses.

In summary, our data provide new insight into the evolutionary history of smallpox vaccines. We suggest that early vaccine strains were related to HPXV and Tian Tan, and that their distribution worldwide originated the two major VACV clades. Thus, HPXV, possibly closely related to the the Beaugency lymph, served as the seed for Dryvax and IOC, whereas a Tian Tan-like population contributed to vaccine clones in the Eurasian clade. We consider that a better understanding of orthopoxvirus evolutionary trajectories may also help shed light into the ongoing MPXV outbreak, as this orthopoxvirus most likely underwent a bottleneck (the transmission that brought it out of Africa) and is probably evolving at accelerated rate^33^.

## Methods

### Sequences, alignments, networks

The complete sequences of VACV genomes were obtained from the NCBI database (https://www.ncbi.nlm.nih.gov/labs/virus/vssi/#/). The number of strains retrieved by NCBI consisted of 71 viruses. Five of these were omitted because they were reassembled genomes^34,35^. Also, two sequences (MG012795 and MG012796) that were previously shown to determine exceptionally long terminal branches in the VACV phylogenetic tree were excluded from the analyses^36^. The final dataset of 64 sequences (Supplementary Table 1) was aligned using MAFFT (v.7.475) with default parameters^37^. SplitsTree4 (v4.16.2)^38^, with HKY85 distances, all polymorphic sites, and without gap sites, was used to generate a neighbor-net split network. Using the same parameters, networks were also generated for 90 CPXV and 50 VARV sequences obtained from NCBI (Supplementary Table 2).

### Linkage disequilibrium and population structure

From the VACV alignment, biallelic parsimony-informative (PI) sites were extracted. In particular, we selected biallelic sites, each with a minimum frequency of two, for those genomic positions where at least 90% of sequences had non-missing information. Gaps and all nonstandard nucleotide bases were considered as missing values. This generated a list of 3794 variants. Some genes in highly dynamic genome regions were not covered by any PI (Supplementary Table 3). To evaluate the level of linkage disequilibrium (LD) in the dataset, the LIAN software was used (v.3.7)^24^. This software tests for independent assortment by computing the number of loci at which each pair of haplotypes differs. Significance was assessed by Monte Carlo simulations (1,000 iterations). The standardized index of association (I_A_^S^) generated by LIAN was associated to the LD intepretation, with zero meaning linkage equilibrium. For the VACV dataset, we obtained a I_A_^S^ of 0.0379, therefore allowing the application of population structure analyses as implemented in the STRUCTURE (v.2.3.4) suite^21^. To run STRUCTURE, we first estimated the allele frequency spectrum parameter (λ) by running the program with *K* = 1, as suggested^22^. The λ parameter was estimated to be equal to 0.8762. Using this value, the linkage model with correlated allele frequencies was performed^22^, estimating K from 1 to 14. In particular, for each K, ten runs were run with a MCMC total chain length of 500,000 iterations and 50,000 iterations as burn-in. The map distances were set equal to PI site physical distances. The optimal K was evaluated using the HARVESTER tool^39^, according to Evanno’s method^40^. The CLUMPAK^41^ software was used to combine replicate runs from the same K and to generate the Q value matrix. The amount of drift that each subpopulation experienced was quantified by the F parameter calculated for the optimal k value^22^. A linkage model analysis run with the optimal K was also performed with the SITEBYSITE option selected, allowing to assign ancestry contribution to every biallelic position for each viral strain.

### PCA

Principal component analysis (PCA) was performed using the same PI matrix used for STRUCTURE. PCA can find patterns in a sample without prior knowledge^42^. The purpose of PCA analysis is to reduce the complexity in high-dimensional data while retaining trends and patterns, by transforming the data into fewer dimensions, which act as summaries of features^42^. PCA was carried out with the mixOmics R package^43^.

### Recombination

Recombination events were analyzed using ClonalFrameML v.1.12^25^. ClonalFrameML requires a sequence alignment and a phylogenetic tree as input files. We used the whole genome alignment as input sequences, and we generated a maximum-likelihood (ML) tree with phyML v.20120412^44^, using a HKY85 substitution model. PhyML also estimates the transition/ transversion ratio; this value, along with the mean branch length, were also provided to ClonalFrameML. A 100 pseudo-bootstrap replicates were performed and R/theta (relative rate of recombination to mutation), 1/delta (inverse mean DNA import length), and nu (mean divergence of imported DNA) were estimated. The r/m parameter (the rate at which nucleotides become substituted as a result of recombination or mutation) was calculated as follow: r/m = delta * R/theta * nu.

### Reporting summary

Further information on research design is available in the Nature Research Reporting Summary linked to this article.

## Supplementary information

Supplementary files

REPORTING SUMMARY

## Data Availability

The list of NCBI IDs of the viral sequences analyzed is provided in Supplementary Table 1–2.
