# Supplementary material for: Genetic ancestry and population structure of vaccinia virus
Source: NPJ Vaccines. 2022 Aug 11;7:92. doi: 10.1038/s41541-022-00519-4 (PMC9372083; doi:10.1038/s41541-022-00519-4)
Supplement: Supplementary file 1 — Supplementary files [file 41541_2022_519_MOESM1_ESM.pdf]

Supplementary Files

Genetic ancestry and population structure of vaccinia virus

Cristian Molteni[1*], Diego Forni[1], Rachele Cagliani[1], Mario Clerici[2,3], Manuela Sironi[1]

[*]corresponding author

[1] IRCCS E. MEDEA, Bioinformatics, Bosisio Parini, Italy

[2] University of Milan, Milan, Italy;

[3] Don C. Gnocchi Foundation ONLUS, IRCCS, Milan, Italy.

Corresponding author: Cristian Molteni, Bioinformatics - Scientific Institute IRCCS E. MEDEA, 23842 Bosisio Parini, Italy. e-mail: cristian.molteni@lanostrafamiglia.it

Supplementary Figures:

Supplementary Figure 1. ΔK analysis

Supplementary Figure 2. Ancestral component probabilities at nucleotide resolution

Supplementary Tables:

Supplementary Table 1. List of analyzed strains

Supplementary Table 2. Cowpox and variola virus strains used for the phylogenetic network analysis

Supplementary Table 3. List of genes that are not or only partially covered by PIs

Supplementary Figure 1. ΔK analysis. Identification of the optimal K for STRUCTURE analysis using the ΔK method.

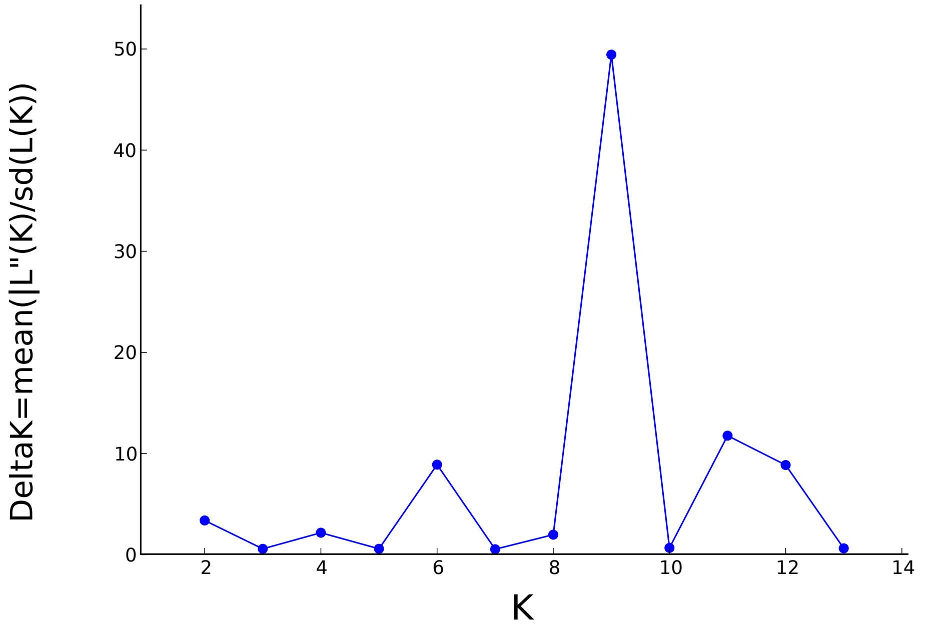

Supplementary Figure 2. Ancestral component probabilites at nucleotide resolution. Each line represents a randomly selected virus for each of the vaccine stocks/groups of VACV; each dot represents a PI site with a probability >0.75 to derive from Lister component. Color is the same as in Figure 3. Positions refer to the genome alignment.

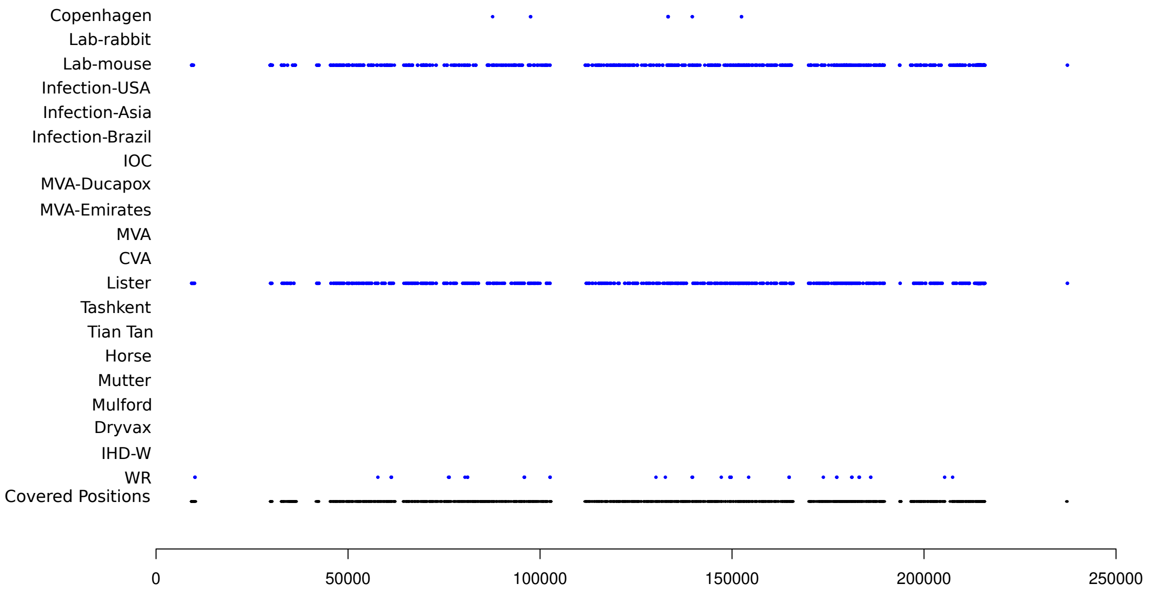

Supplementary Table 1. List of analyzed strains.

Accession	Strain	Vaccine name/Type
M35027	Copenhagen	Copenaghen
AM501482	CVA (parental virus of MVA)	CVA
AY313847	acambis clone 2000	Dryvax
DQ377945	3737	Dryvax
JN654985	DPP20	Dryvax
JN654979	DPP12	Dryvax
JN654981	DDP15	Dryvax
JN654982	DPP16	Dryvax
JN654983	DPP17	Dryvax
JN654978	DPP11	Dryvax
JN654976	DPP9	Dryvax
JN654977	DPP10	Dryvax
AY313848	acambis clone 3	Dryvax
JN654984	DPP19	Dryvax
KJ125438	DPP25	Dryvax
JN654986	DPP21	Dryvax
DQ439815	Duke	Dryvax
JN654980	DDP13	Dryvax
DQ792504	MNR-76	HPXV
KJ125439	IDH-W1	IHD-W
KC201194	IDH-W	IHD-W
MG599038	Karachi 2005	Infection-Asia
MK239753	NIV143262	Infection-Asia
MK239754	NIV116233	Infection-Asia
MK239755	NIV116234	Infection-Asia
MW883890	BPXV/buffalo/India/2011/ Meerut-1/Control/P40	Infection-Asia
MW883891	BPXV/buffalo/India/2011/ Meerut-1/CGP57380/P40	Infection-Asia
MW883892	BPXV/buffalo/India/2011/ Meerut-1/P0	Infection-Asia
KT013210	Cantagalo virus CM01	Infection-Brazil
MW018153	Cantagalo MI233	Infection-Brazil
MW018154	Cantagalo VI04	Infection-Brazil
MW018155	Cantagalo CG04	Infection-Brazil
MW018156	Cantagalo ALEH2	Infection-Brazil
KF179385	Serro 2	Infection-Brazil
MT227314	Acambis/2019	Infection-USA
KT184691	VACV-IOC-B388	IOC
KT184690	VACV-IOC-B141	IOC
KF866253	WAU86/88-1	Lab-mouse
AY484669	Rabbitpox	Lab-rabbit
KX061501	Butantan	Lister
EU410304	GLV-1h68	Lister
DQ121394	VACV107	Lister
AY678276	VACV-LO	Lister

AY678275	LC16m8	Lister
AY678277	LC16m0	Lister
MF477237	Mulford 1902	Mulford
MN369532	VK01	Mutter
AY603355	acambis clone 3000	MVA
U94848	MVA	MVA
DQ983236	MVA-1721	MVA
DQ983237	MVA-572	MVA
DQ983238	MVA-BN	MVA
MT648498	Ducapox vaccine (batch number DPV0816)	MVA-Ducapox
MT946551	Ducapox vaccine (batch number DPV0818)	MVA-Emirates
KM044309	TK3	Tashkent
KM044310	TK4	Tashkent
JX489136	TT09	Tian Tan
KC207811	TP05	Tian Tan
JX489135	TT08	Tian Tan
JX489137	TT10	Tian Tan
JX489138	TT11	Tian Tan
JX489139	TT12	Tian Tan
KC207810	TP03	Tian Tan
NC_006998	WR	WR

Supplementary Table 2. Cowpox and variola virus strains used for the phylogenetic network analysis.

Accession	Species
MK035746	cowpox virus
KC813508	cowpox virus
LN864565	cowpox virus
MK035747	cowpox virus
KC813501	cowpox virus
KC813503	cowpox virus
KC813505	cowpox virus
KC813512	cowpox virus
MK035748	cowpox virus
MK035750	cowpox virus
MK035753	cowpox virus
MK035757	cowpox virus
MK035759	cowpox virus
MK035749	cowpox virus
KC813510	cowpox virus
HQ420895	cowpox virus
MK035751	cowpox virus
MK035758	cowpox virus
KY549146	cowpox virus
KY549147	cowpox virus
MK035755	cowpox virus
MK035754	cowpox virus
MK035756	cowpox virus
KC813495	cowpox virus
LT896719	cowpox virus
MK035752	cowpox virus
KC813502	cowpox virus
KC813509	cowpox virus
KC813491	cowpox virus
LT993231	cowpox virus
LT896728	cowpox virus
LT896723	cowpox virus
LT993230	cowpox virus
KC813506	cowpox virus
LT993228	cowpox virus
LN879483	cowpox virus
LT896726	cowpox virus
HQ420898	cowpox virus
LT993232	cowpox virus
LT896733	cowpox virus
LT896718	cowpox virus

KC813507	cowpox virus
KC813511	cowpox virus
LT896732	cowpox virus
LT896720	cowpox virus
LT896729	cowpox virus
LT896731	cowpox virus
KC813496	cowpox virus
LT896725	cowpox virus
LT896727	cowpox virus
LT896730	cowpox virus
KC813504	cowpox virus
KC813497	cowpox virus
KC813492	cowpox virus
KC813498	cowpox virus
KC813500	cowpox virus
KY463519	cowpox virus
KY569020	cowpox virus
KY569022	cowpox virus
KY569021	cowpox virus
KY569018	cowpox virus
KY569019	cowpox virus
KC813494	cowpox virus
HQ420897	cowpox virus
HQ420894	cowpox virus
KY549149	cowpox virus
HQ420896	cowpox virus
KY549144	cowpox virus
LT896724	cowpox virus
KY549145	cowpox virus
KC813499	cowpox virus
LN864566	cowpox virus
HQ420899	cowpox virus
KY549150	cowpox virus
LT896722	cowpox virus
KY549148	cowpox virus
HQ420900	cowpox virus
LT883663	cowpox virus
DQ437593	cowpox virus
LT993226	cowpox virus
NC_003663	cowpox virus
KY549151	cowpox virus
KC813493	cowpox virus
KY549143	cowpox virus
HQ407377	cowpox virus
HQ420893	cowpox virus

X94355	cowpox virus
KY369926	cowpox virus
LT896721	cowpox virus
LR812035	cowpox virus
P328	variola virus
KY358055	variola virus
DQ437584	variola virus
DQ441416	variola virus
DQ441426	variola virus
DQ441437	variola virus
DQ441434	variola virus
DQ441447	variola virus
DQ441419	variola virus
Y16780	variola virus
DQ437590	variola virus
NC_001611	variola virus
DQ441432	variola virus
DQ437582	variola virus
DQ441430	variola virus
DQ441444	variola virus
DQ441442	variola virus
DQ437591	variola virus
DQ441428	variola virus
DQ441429	variola virus
DQ441431	variola virus
DQ441433	variola virus
DQ441446	variola virus
DQ441445	variola virus
DQ437580	variola virus
DQ437581	variola virus
DQ437583	variola virus
DQ437585	variola virus
DQ437586	variola virus
DQ437587	variola virus
DQ437588	variola virus
DQ437589	variola virus
DQ437592	variola virus
DQ441417	variola virus
DQ441418	variola virus
DQ441420	variola virus
DQ441421	variola virus
DQ441422	variola virus
DQ441423	variola virus
DQ441427	variola virus
DQ441435	variola virus

DQ441436	variola virus
DQ441443	variola virus
DQ441448	variola virus
DQ441424	variola virus
DQ441425	variola virus
DQ441438	variola virus
DQ441439	variola virus
DQ441440	variola virus
DQ441441	variola virus

Supplementary Table 3. List of genes that are not or only partially covered by PIs.

Alignment start	Alignment end	VACV WR start	VACV WR end	Annotated genes
1	9137	1	4045	VACWR001^
10398	29711	5192	10359	VACWR004^, VACWR005, VACWR006, VACWR007,VACWR008^, VACWR009^, VACWR010, VACWR011^, VACWR012
30272	32557	10920	13096	VACWR014, VACWR015, VACWR016, VACWR017, VACWR018
36607	41591	17066	22023	VACWR023, VACWR024, VACWR025, VACWR026, VACWR027, VACWR028
42462	45266	22893	25673	VACWR030, VACWR031, VACWR032
62320	64313	NA	NA	Foreign insertion in EU410304.1
102929	111609	NA	NA	Foreign insertion in EU410304.1
165996	169922	135196	139050	VACWR147, VACWR148
189812	193610	158585	162186	VACWR178, VACWR179, VACWR180
194085	196514	NA	NA	Foreign insertion in EU410304.1
205480	206717	NA	NA	Only present in MN369532
215843	237078	171646	189950	VACWR192, VACWR193, VACWR194, VACWR195, VACWR196, VACWR197, VACWR198, VACWR199, VACWR200, VACWR201, VACWR202, VACWR203, VACWR204, VACWR204.5, VACWR205, VACWR206, VACWR207, VACWR208^, VACWR209^, VACWR210^, VACWR211^, VACWR212, VACWR213, VACWR214, VACWR215^, VACWR216, VACWR217
237344	246283	190178	194711	VACWR218^

^ Denotes genes within inverted terminal repeats (ITRs).
